# Serum as an alternative matrix for reliable molecular diagnosis of fowl adenovirus infections

**DOI:** 10.3389/fvets.2026.1800365

**Published:** 2026-05-22

**Authors:** Amina Kardoudi, Kichou Faouzi, Ikram Ouchhour, Benani Abdelouaheb, Fellahi Siham

**Affiliations:** 1 Department of Veterinary Pathology and Public Health, Hassan Institut Agronomique et Vétérinaire Hassan II, Rabat, Morocco; 2 Molecular Biology Laboratory, Medical Biology Department, Pasteur Institute of Morocco, Casablanca, Morocco

**Keywords:** cloacal swab, fowl adenovirus, gizzard, liver, penton gene, real-time PCR, sample type, serum

## Abstract

Fowl aviadenoviruses (FAdVs) are major pathogens of poultry and are associated with economically important diseases highlighting the need for reliable molecular diagnostic and practical sample matrices suitable for routine diagnosis and large-scale surveillance. In this study, we developed a universal real-time PCR assay targeting a conserved region of the FAdV penton gene and evaluated serum as a reliable matrix for molecular diagnosis. The penton-based real-time PCR assay exhibited excellent analytical performance, showing strong linearity over a wide dynamic range (10^2^–10^6^ copies/μL), a high amplification efficiency of 97%, and a limit of detection of 5 copies/μL. The inter-specificity of the assay was evaluated using Avian Influenza Virus (AIV), Newcastle Disease Virus (NDV), Infectious Bronchitis Virus (IBV), and Infectious Laryngotracheitis Virus (ILTV). Clinical performance was assessed by quantitative comparison with a reference universal real-time PCR assay targeting the 52 K gene. Analysis of 56 field samples representing four circulating FAdV serotypes revealed excellent agreement between the two assays (R^2^ = 0.9762). The diagnostic performance of serum was further investigated using 320 experimental samples collected from chickens experimentally inoculated with FAdV-1 and FAdV-8a and monitored over a 30-day period. Serum, gizzard, liver, and cloacal swab samples were collected longitudinally, and viral loads were quantified in each matrix. Group-based correlation analyses revealed distinct, biologically consistent patterns depending on the infecting serotype. In groups infected with FAdV-1, serum viral loads showed strong correlations with gizzard samples. In contrast, in the FAdV-8a infected group, elevated viral loads were observed in both liver and gizzard samples, highlighting the dual hepatic and gastrointestinal tropism and the higher pathogenicity of this serotype. Temporal analyses further demonstrated a progressive redistribution of viral loads, characterized by declining hepatic viral loads and increasing viral detection in serum and cloacal swabs at later stages of infection, indicating systemic dissemination.

## 1 Introduction

Fowl adenoviruses (FAdVs) are non-enveloped double-stranded DNA viruses that infect domestic poultry and wild birds. Based on genomic characterization and virus neutralization assays, FAdVs are classified into five species (FAdV-A to FAdV-E) encompassing twelve serotypes (1–7, 8a, 8b, 9–11) (1, 2). Although many FAdV infections are subclinical, several serotypes are associated with clinically significant diseases, including inclusion body hepatitis (IBH) (3, 4), adenoviral gizzard erosion (AGE) (5, 6), and hydropericardium-hepatitis syndrome (HHS) (7), which result in substantial economic losses in the poultry industry due to increased mortality, impaired growth performance, and reduced production efficiency (6).

FAdV-associated diseases have been reported worldwide, highlighting the global distribution and epidemiological importance of these viruses (7–13). In Morocco, outbreaks of IBH and AGE have been documented and associated with FAdV serotypes 8b/11 and 1/8a, respectively (3, 14–16). These findings underline the need for reliable molecular diagnostic tools to support timely detection, surveillance, and effective management of FAdV infections in poultry production systems.

Conventional diagnosis of FAdV infections relies on virus isolation followed by electron microscopy or molecular characterization techniques (17–20). While informative, these approaches are labor-intensive, time-consuming, and dependent on sample quality and viral viability, limiting their routine diagnostic applicability (19, 21). Conventional PCR assays targeting the hexon gene are widely used for FAdV detection; however, genotyping typically requires additional post-PCR procedures such as restriction enzyme digestion or nucleotide sequencing, which increase turnaround time and laboratory workload (9, 12, 22).

Real-time PCR (qPCR) assays have therefore become the method of choice for FAdV diagnosis due to their high sensitivity, rapid turnaround time, and quantitative capabilities. Universal real-time PCR assays based on SYBR Green or probe-based chemistries have been reported, demonstrating improved analytical performance compared with conventional PCR (23–26). Nevertheless, most molecular diagnostic approaches for FAdV infections continue to rely primarily on tissue-based samples, such as liver, gizzard, or cloacal swabs.

In routine molecular diagnosis of fowl adenovirus infections, tissue samples such as liver, gizzard, or cloacal swabs are commonly used (14, 15, 27). However, the processing of these matrices requires multiple handling steps, including tissue dissection, homogenization, and selection of representative fragments, which may introduce variability in sample preparation and turnaround time. In addition, tissue-based testing is inherently influenced by heterogeneous viral distribution within organs and by differences in the amount of biological material used for nucleic acid extraction (20, 28).

Serum represents a biologically relevant matrix for molecular investigations, as it reflects the systemic circulation of viral genomes during infection. From a methodological perspective, serum offers a simplified and standardized workflow based on the use of a defined input volume, which facilitates harmonization of DNA extraction and quantitative analysis across samples (29–31). These characteristics make serum particularly attractive for large-scale diagnostic applications, longitudinal monitoring, and comparative studies, where consistency, reproducibility, and processing efficiency are critical. Despite these potential advantages, the suitability of serum for molecular detection and quantification of FAdV DNA has not been systematically evaluated.

In this context, the present study aimed to develop and validate a universal TaqMan-based real-time PCR assay targeting a conserved region of the FAdV penton gene and to evaluate the potential of serum as a reliable alternative matrix for molecular diagnosis of FAdV infections using both field samples and controlled experimental infections.

## 2 Materials and methods

### 2.1 Development and validation of universal real-time PCR assay

#### 2.1.1 Ethical statement

Tissue used in this study were collected from naturally dead birds that were clinically sick from commercial broilers and layer flocks. The protocol was applied in accordance with Moroccan legislations on laboratory animal care and use and animal protocols approved by the institutional Ethical Committee for Animal Veterinary Science and Public health (CESASPV) and with international standards cited in numerous scientific references and the WOAH Manual (2024) [World Organization of Animal Health (Terrestrial Manual Online Access, s. d. WOAH)].

#### 2.1.2 References strain and clinical sample

Standard FAdV strains were obtained from the University of Veterinary Medicine, Vienna (Vetmeduni, Vienna, Austria), provided on FTA cards specific to each serotype. Fragments from the impregnated sections of each card were cut using sterile scissors, placed in individual Eppendorf tubes, and washed separately with Tris-EDTA buffer. The reference FAdV strains and their GenBank accession numbers used in this study are listed in Table 1.

**Table 1:** References FAdV strains and corresponding access number used in this study.

Serotype	Strain name	Access number
FAdV-1	CELO	MK572875.1
FAdV-2	658	KT862806.1
FAdV-3	SR49	KT862807.1
FAdV-4	KR5	NC_075488.1
FAdV-5	340	NC_021221.1
FAdV-6	CR119	NC_038332.1
FAdV-7	YR36	KT862809.1
FAdV-8a	TR59	KT862810.1
FAdV-8b	764	KT862811.1
FAdV-9	A2-A	AF083975.2
FAdV-10	C2B	MK572851.1
FAdV-11	380	KT862812.1

A total of 65 clinical samples, including liver, and gizzard were collected from broiler and layer chicken farms suspected of IBH or AGE in various regions of Morocco. The samples were homogenized, and viral DNA was extracted according to the protocols described by Abghour et al. (3) and Ouchhour et al. (14) for subsequent molecular characterization.

#### 2.1.3 Primer design

Whole genome sequences of 12 FAdV reference strains, each representing a distinct serotype, were downloaded from the NCBI database and aligned using the ClustalW algorithm by Geneious Prime 2024.0.2 software. Additional publicly available FAdV sequences from NCBI, representing diverse serotypes, were also included to further confirm the conservation of the primer binding regions. A highly conserved region within the FAdV penton gene was identified and selected for primer and probe design using Geneious Prime 2024.0.2 and Primer3 software. To ensure specificity, the designed primers and probe were evaluated using the BLAST tool. Degenerate bases were incorporated to include sequence variations among different serotypes. All primers and the probe were synthesized by Sangon Biotech (Shanghai, China).

#### 2.1.4 Viral DNA extraction

Viral DNA from FAdV reference strainswas extracted using the MagPurix EVO Automated Nucleic Acid Purification System (Zinexts, Taiwan) with the MagPurix^®^ Viral/Pathogen Nucleic Acid Extraction Kit, following the manufacturer’s instructions. In contrast, viral DNA fromfield samples was extracted using the Kylt^®^ RNA/DNA Purification Kit (Hoeltinghausen, Germany), also according to the manufacturer’s protocol.

#### 2.1.5 Real time PCR

DNA extracted from field samples was initially amplified using a universal real-time PCR assay targeting the 52 K gene, which served as a reference test in this study. The primers used were 52 K-fw (sense): 5′ ATGGCK CAG ATG GCY AAG G 3′ and 52 K-rv (antisense): 5′ AGC GCC TGG GTC AAA CCG A 3′ (Table 2). Real-time PCR was performed on a QuantStudio 5 Real-Time PCR System (Thermo Fisher Scientific, USA). Each 20 μL reaction mixture consisted of 5 μL of DNA and 15 μL of a reaction mix containing 10 μL of the SensiFAST SYBR Lo-Rox Mix (Bioline, New York, NY), which includes Taq DNA polymerase, SYBR Green I, and a deoxynucleotide triphosphate mix, along with 0.8 μL of each primer (52 K-fw/52 K-rv) at a concentration of 10 μM. PCR cycling conditions were performed as described by (23), ensuring optimal amplification specificity and efficiency.

**Table 2:** Primers and probes used in this study.

Primer/probe name	Primer position	Sequence (5′…0.3′)	References
52 K-fw	13,075–13,093	ATGGCK CAG ATG GCY AAG G	Günes et al. (23)
52 K-rv	13,250–13,232	AGC GCC TGG GTC AAACCG A	
Uni-FAdV-F2	16,419–16,437	CCGCYTTYAACCGSTTTCC	This study
Uni-FAdV-R2	16,508–16,490	GGAAACGGTTAAGCGG	

Position is given for the FAdV-1 CELO complete genome, (GenBank accession number: AAU46933).

#### 2.1.6 Universal real-time PCR test optimization and standard curve

To optimize the PCR reaction, different concentrations of primers and probe were tested using a reaction mixture composed of 5 μL of 4X CAPITAL™ 1-Step qRT-PCR Green Master Mix (New England Biolabs, USA), 5 μL of DNA, and nuclease-free water to a final volume of 20 μL. The assay was performed on the LightCycler z480 real-time PCR system (Roche Diagnostics, USA). FAdV quantification was carried out using the following thermal cycling profile: reverse transcription at 50 °C for 10 min, an initial activation at 95 °C for 3 min, followed by 35–40 amplification cycles consisting of denaturation at 95 °C for 10 s and an annealing/extension step at 60–68 °C for 30 s.

A positive control corresponding to FAdV-11, with a concentration of 6 Log_10_UI/ml, was used to generate a standard curve through a series of 1:10 dilutions. Each dilution was analyzed in triplicate within the same reaction, and the results were processed using Light Cycler software, version 1.5.0.

#### 2.1.7 Sensitivity analysis

The sensitivity of the assay was evaluated using a ten-fold serial dilution of FAdV-11 DNAranging from 10^2^ to 10^6^ copies/mL. Each dilution was tested in triplicate using the universal real-time FAdV PCR assay to determine the lowest concentration reliably detected. To confirm the assay’s detection limit, the dilutions corresponding to 10^2^, 10, and 5 copies/mL were tested in triplicate separately within a single run.

#### 2.1.8 Specificity analysis

To assess the specificity of the universal real-time PCR assay,12 FAdV reference strains was tested: CELO (FAdV-1), SR48 (FAdV-2), SR49 (FAdV-3), KR5 (FAdV-4), 340 (FAdV-5), CR119 (FAdV-6), YR36 (FAdV-7), TR59 (FAdV-8a), 764 (FAdV-8b), A2-A (FAdV-9), C2B (FAdV-10), and 380 (FAdV-11) (Table 1). In addition, cDNA samples from other avian viruses, including Avian Influenza Virus (AIV), Newcastle Disease Virus (NDV), Infectious Bronchitis Virus (IBV), and Infectious Laryngotracheitis Virus (ILTV), were tested to confirm assay’s specificity and exclude cross-reactivity.

#### 2.1.9 Quantification and unit conversion

To ensure consistency and clarity in data presentation, all quantitative results were expressed as Log₁₀ copies/mL, which were considered equivalent to Log₁₀ International Unit (IU)/mL, since 1 International Unit (IU) approximately corresponds to 1 genome copy based. For concentrations lower than 10 copies/mL, values were expressed in copies/mL rather than in logarithmic form, to simplify data visualization and improve the interpretation of results, especially near the assay’s detection limit.

### 2.2 Serum’s test

#### 2.2.1 Chickens and ethics statement

All animal research conducted in this study received ethical approval for sampling and manipulation from the institutional Ethical Committee for Animal Veterinary Science and Public health (CESASPV). The protocol was applied in accordance with international standards cited in numerous scientific references and the OIE Manual (2015) titled “Manual of diagnostic tests and vaccines for terrestrial animals.

Birds were identified individually by wing banding and randomly assigned to six groups of 20 birds/group. All experiments were conducted in a negative-pressure Biosafety Level 3 room; the birds were reared in separate rooms and had ad libitum access to water and a starter diet.

#### 2.2.2 Experimental design

**Figure 1:**
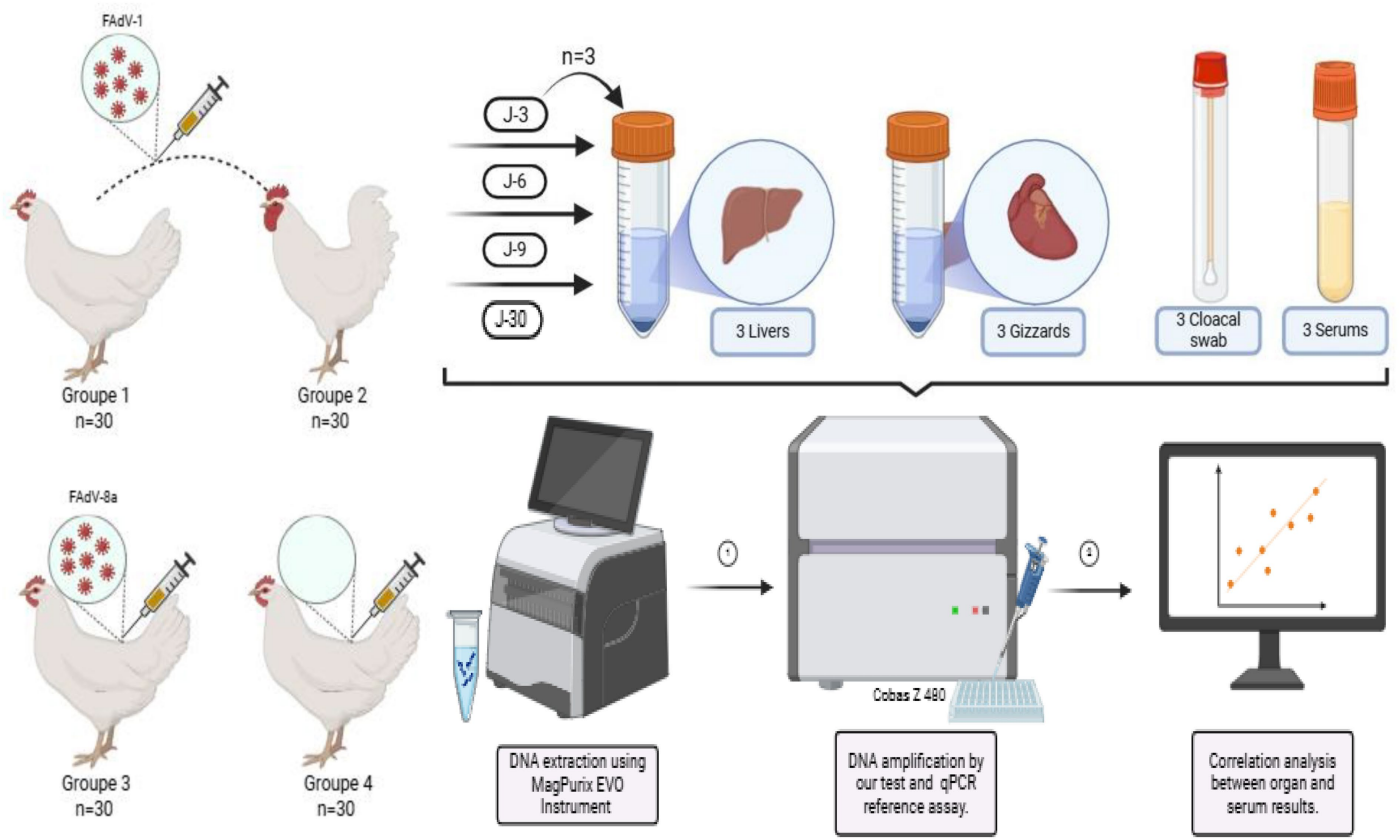
Schematic overview of the experimental workflow. Four groups of chickens (*n* = 30 per group) were inoculated with either FAdV-1 or FAdV-8a, while control groups received no virus. Organ sampling (liver and gizzard), cloacal swabs, and serum samples were collected at days 3, 6, 9, 12, 15, 18, 21, 24, 27, and 30 post-infection (three chickens randomly selected per time point). Viral DNA was extracted using the MagPurix EVO instrument and amplified using both the newly developed qPCR assay and the reference test on the Cobas z480 system. Viral loads obtained from organs and serum samples were then compared through correlation analysis.

A total of one hundred twenty specific-pathogen-free (SPF) White Leghorn egg-laying hens (Green Biological Engineering Co., Yangling, China) were housed in isolators under negative pressure with food and water provided ad libitum. They were randomly divided into control groups and 3 experimental groups of 30 birds. Group 1 and 3 were orally inoculated with Moroccan isolate corresponding to FAdV-8a and FAdV-1, respectively, at 10 days of age, while the birds in group 2 were challenged at 35 days of age with FAdV-1. Finally, group 4 contained the negative control flock, which was inoculated with phosphate-buffered saline via the same route. At each sampling point (3, 6, 9, 12, 15, 18, 21, 24, 27, and 30 days post-infection, dpi), three chickens from each group were randomly selected and euthanized by intravenous injection of pentobarbital sodium solution (200 mg/mL) *Dolethal* (pentobarbital sodium 200 mg/mL solution for injection; Vetoquinol). Following euthanasia, gizzard, liver, blood, and cloacal swab samples were collected. Cloacal swabs were placed in 1 mL of antibiotic-phosphate-buffered saline (PBS) containing 1 mg/mL streptomycin and 100,000 IU/mL penicillin. Blood samples were collected in plain tubes without anticoagulant, and centrifuged to obtain serum, which was transferred into sterile tubes. The organs from each selected chicken were also placed in sterile tubes. All samples were stored at −20 °C until further DNA extraction (Figure 1).

#### 2.2.3 DNA extraction and real-time PCR

Viral DNA from collected organs and serums were extracted using the MagPurix EVO Automated Nucleic Acid Purification System (Zinexts, Taiwan) with the MagPurix^®^ Viral/Pathogen Nucleic Acid Extraction Kit, following the manufacturer’s instructions. DNA extracted from field samples was amplified using both the universal real-time PCR assay targeting the 52 K gene (reference method) following previously described protocols in 1.4 paragraph, and the newly developed universal real-time PCR/Penton designed in this study.

#### 2.2.4 Statistical analysis

Statistical analyses were conducted to evaluate the correlation between viral loads obtained from serum and other sample types, including liver, gizzard, and cloacal swabs, within each experimental group. The ability of serum samples to reflect and trace viral load dynamics from 3 to 30 days post-infection (dpi) was also assessed and compared with the other sample types. Linear regression analysis was performed using Microsoft Excel.

## 3 Results

### 3.1 Primers design

**Figure 2:**
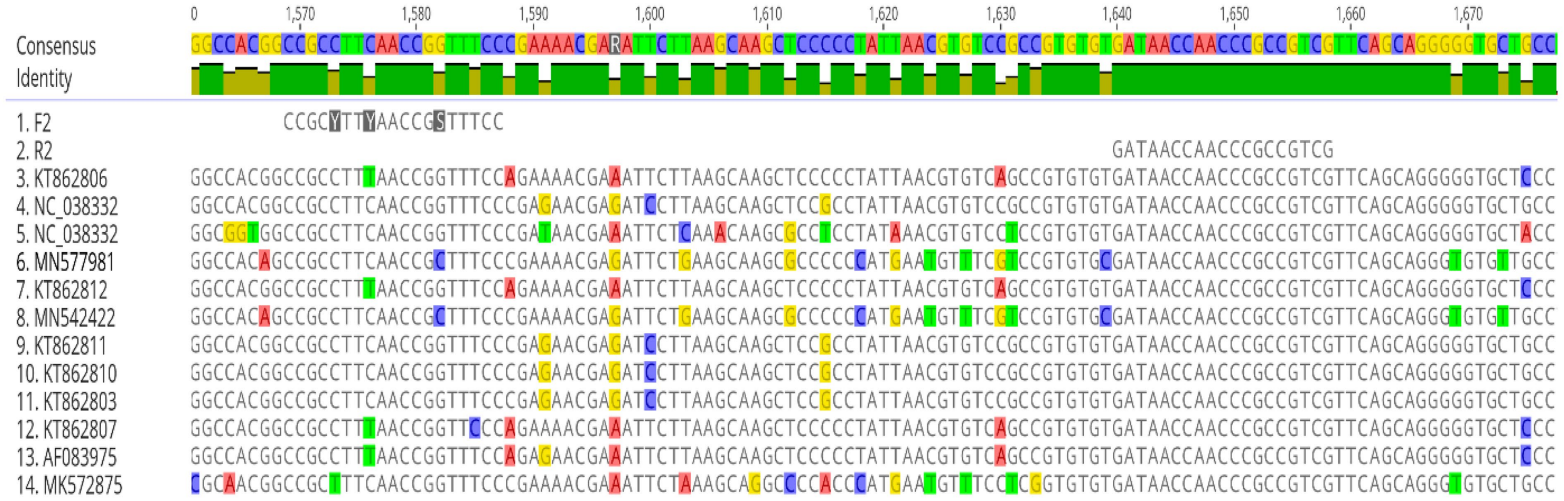
Multiple sequence alignment of the conserved penton gene region used for primer and probe design. Alignment of twelve reference FAdV serotypes showing the highly conserved region selected as the target for the universal assay. The forward primer, reverse primer are shown, with degenerated bases incorporated in the forward primer to accommodate inter-serotype variability.

The complete genomes of twelve reference Fowl Aviadenovirus (FAdV) strains were aligned using the ClustalW algorithm implemented in Geneious Prime. The alignment revealed a highly conserved region within the penton gene shared among all FAdV serotypes. This region was selected as the target for primer and probe design because of its high level of conservation across multiple FAdV serotypes, making it a suitable candidate for the development of a universal detection assay. The primers and probe were initially designed using the Primer3 algorithm, and their specificity, and potential secondary structures were subsequently verified and optimized using Geneious Prime 2024.0.2 The designed primer set amplifies a 89 bp fragment within the conserved pentongene. The sequences of the primers along with other primers used in this study, are listed in Table 2. Figure 2 shows the alignment of these primers with reference strains corresponding to the twelve FAdV serotypes.

### 3.2 Linearity test

**Figure 3:**
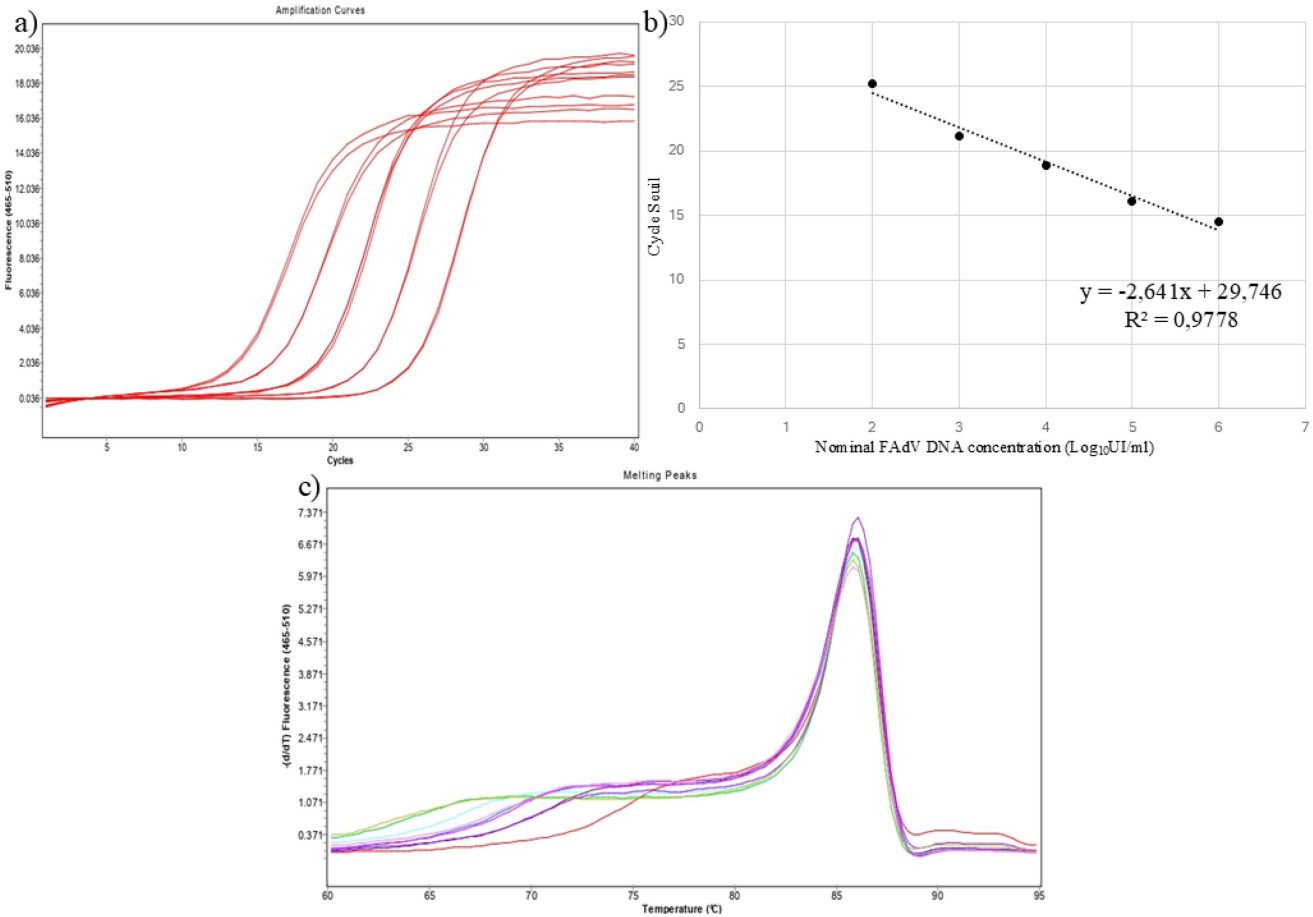
**(a)** Amplification curves from a 10-fold dilution series of FAdV DNA for ranging from 1 to 6 Log_10_UI/ml. **(b)** Standard curve of the universal real-time PCR assay. This curve was generated by plotting mean Ct values from three independent reactions against the logarithmic concentrations of FAdV. **(c)** Melting curves corresponding to each dilution.

The linearity of the assay was assessed using a panel of tenfold serial dilutions of FAdV-11 genomic DNA, ranging from 10^2^ to 10^6^ copies/μL, each tested in triplicate. Mean Ct values for each dilution were used to generate the standard curve. The assay demonstrated strong linearity over the 10^2^–10^6^ range (Figure 3a), with a regression coefficient (R^2^) of 0.9551 and an amplification efficiency of 97%. The equation of the standard curve was y = −2.641x + 29.746 (Figure 3b). In addition, the corresponding HRM profile generated from the dilution series showed a single, overlapping melting peak across all concentrations, each represented by a distinct color (Figure 3c). This consistent melting behavior confirms the assay’s high specificity, as no secondary or nonspecific peaks were observed (Figure 3c).

### 3.3 Specificity test

**Figure 4:**
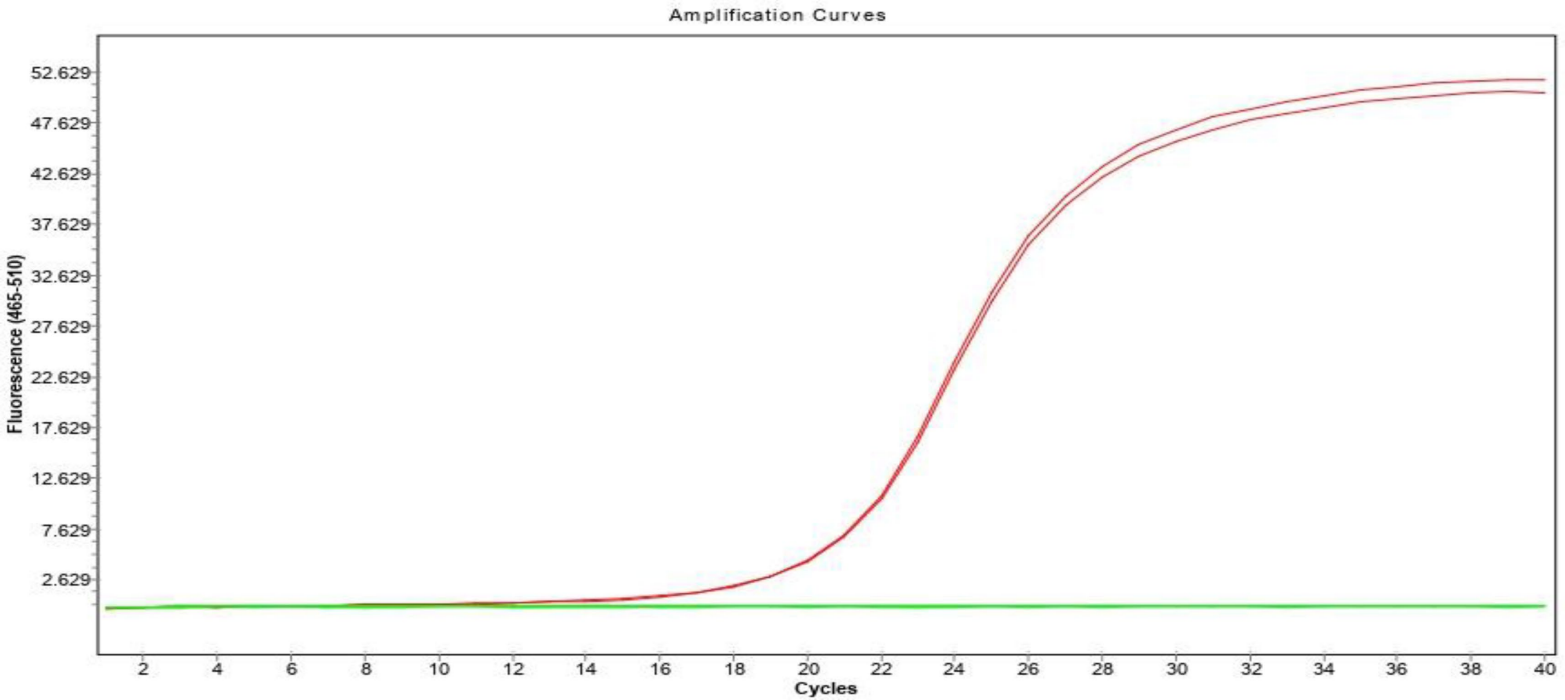
Specificity analysis of the universal FAdV qPCR assay. Amplification curves obtained from duplicate FAdV samples (red) compared to non-amplifying curves from AIV H9N2, NDV, IBV, and ILTV (green). Only FAdV generated a detectable signal, confirming the high specificity and absence of cross-reactivity with other avian pathogens.

The inter-specificity of the assay was evaluated using cDNA from four avian viruses: AIV H9N2 subtype, NDV, IBV, ILTV. No amplification was observed for any of these viruses, confirming the assay’s high specificity for FAdV. Only the FAdV samples, tested in duplicate, showed clear amplification curves, while all non-FAdV samples remained flat throughout the reaction (Figure 4). These results demonstrate that the test is selective for FAdV and does not cross-react with other common avian pathogens. This high level of specificity ensures the accuracy of the assay in differentiating FAdV infections from other avian viral diseases.

### 3.4 Sensitivity test

**Figure 5:**
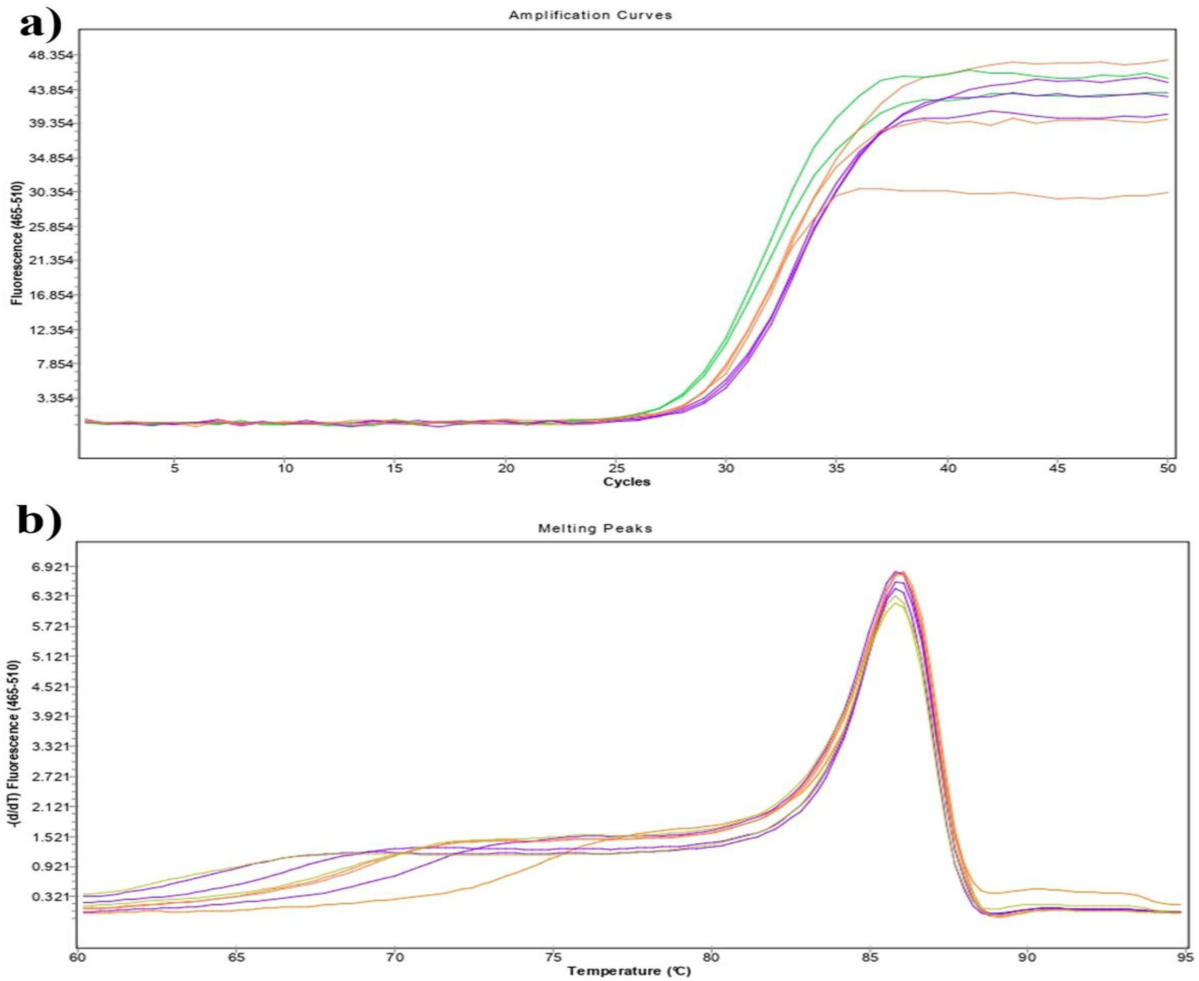
**(a)** Sensitivity assessment of the universal real-time PCR assay using low-copy templates. Amplification curves are shown for dilutions containing 102 (Green), 10 (Orange), and 5 copies/mL (Purple) of FAdV DNA, demonstrating the assay’s ability to reliably detect and amplify very low template concentrations near the limit of detection. **(b)** Melting curves corresponding to each dilution.

A serial dilution ranging from 10^6^ to 10 copies/mL was analyzed to determine the limit of detection (LOD) of the assay. The test consistently detected as low as 10 copies/ml with a confidence level of ≥ 95%. To validate these results, the dilution corresponding to 10^2^, 10, and 5 copies/mL were tested in triplicate in the same reaction over three successive days, confirming the assay’s sensitivity (Figure 5a). The melting curves corresponding to each dilution used were captured and presented in Figure 5b.

### 3.5 Clinical performance

#### 3.5.1 Concordance with universal real-time PCR/52 K test

**Figure 6:**
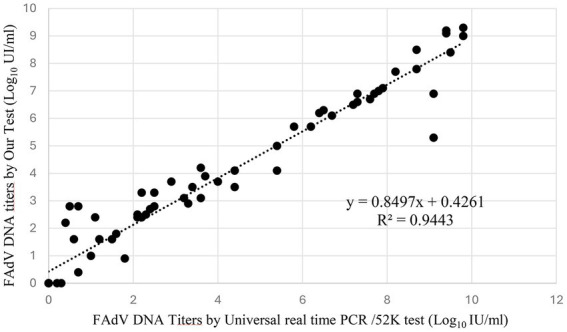
Linear regression analysis of correlation between our universal real-time/penton test and universal real-time PCR/52 K test.

To assess the clinical performance of the assay, a total of 56 FAdV-positive field isolates representing four circulating FAdV serotypes were analyzed. Among these, FAdV-8a (2/56) and FAdV-1 (9/56) were isolated from cases of adenoviral gizzard erosion, while FAdV-8b (18/56), FAdV-11 (22/56), and FAdV-D (5/56) originated from outbreaks of inclusion body hepatitis. All samples were tested in parallel using the newly developed universal real-time PCR assay and the reference 52 K real-time PCR assay described by Günes et al. (23). To enable quantitative comparison, Ct values obtained from both assays were converted to log₁₀ IU/mL. All FAdV-positive field isolates were detected, confirming the high diagnostic accuracy of the assay. Linear regression analysis performed on all tested samples (*n* = 56) demonstrated a strong quantitative agreement between the two methods, described by the equation y = 0.8549x + 0.6032 and a coefficient of determination of R^2^ = 0.9443 (Figure 6).

#### 3.5.2 Concordance with universal real-time PCR/52 K testAcross four sample types

To further evaluate the analytical performance of our assay, a total of 320 experimental samples were analyzed, representing four types of matricesserum, cloacal swabs, liver, and gizzard collected from chickens experimentally infected with FAdV-1 or FAdV-8a. All samples were tested in parallel using the reference Universal Real-Time PCR/52 K assay and our newly developed universal assay. The viral loads obtained from both methods were compared to assess quantitative concordance.

**Figure 7:**
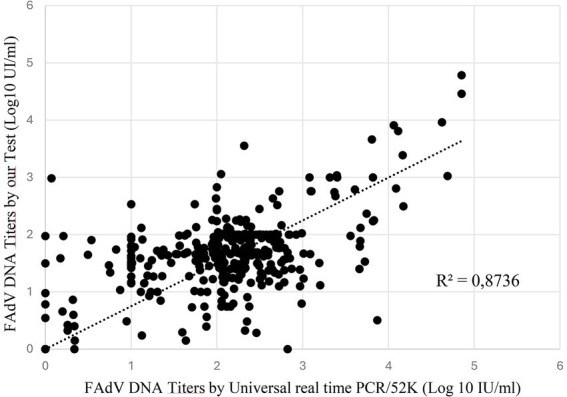
Linear regression analysis of the correlation between the universal real-time Penton assay and the universal real-time PCR/52 K assay performed on 320 samples distributed across four sample types.

A scatter plot was generated and fitted with a linear trendline, for which the interception and slope were determined. The regression analysis revealed a clear positive correlation between the two assays across all sample types and serotypes, with a coefficient of determination (R^2^ = 0.8736) (Figure 7). This strong correlation indicates that both assays exhibit comparable quantitative performance. Overall, these results demonstrate that our universal PCR assay provides reliable, consistent, and concordant viral load measurements relative to the reference method, regardless of sample origin or FAdV serotype.

**Figure 8:**
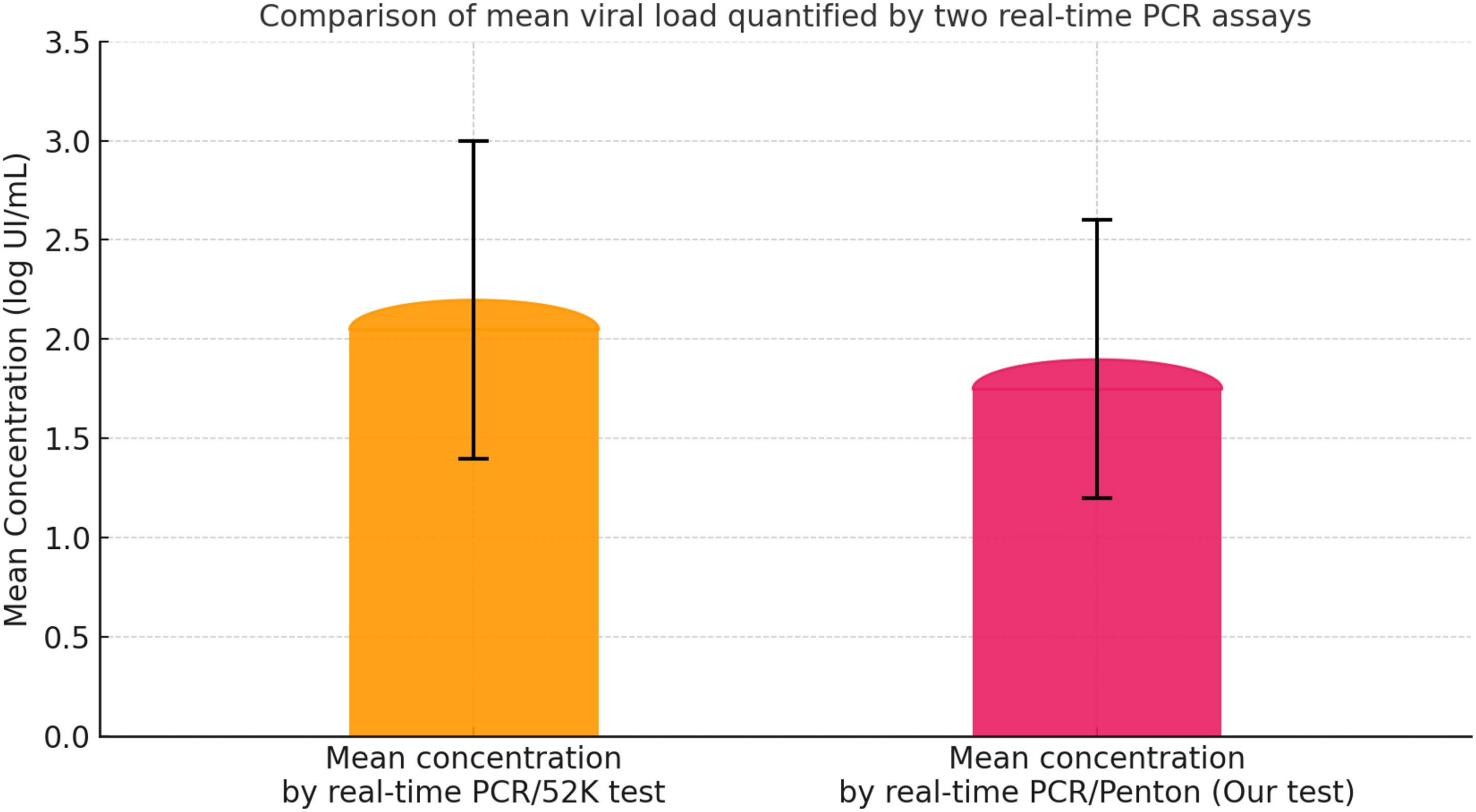
Comparison of mean viral loads measured by the two real-time PCR assays. Bar plot showing the mean log₁₀ IU/mL concentration quantified by the universal real-time PCR/52 K assay (orange) and the new penton-based assay (pink) across 320 experimental samples derived from serum, cloacal swabs, liver, and gizzard. Error bars represent standard deviations. The close similarity between the two mean values indicates that both assays provide consistent and comparable quantification of FAdV DNA.

To complement the regression and agreement analyses, the mean viral loads quantified by both assays were compared across all 320 experimental samples. As shown in Figure 8, the mean concentration obtained with reference Universal Real-Time PCR/52 K assay was slightly higher than that obtained with our penton-based assay, although the two means remained within overlapping error margins (Figure 8). This overlap indicates that both assays provide comparable quantitative estimates of FAdV DNA levels. The dispersion reflected by the error bars further confirms that the variability between the two methods is minimal and does not significantly affect overall quantification performance.

### 3.6 Evaluation of serum samples as an alternative for rapid FAdV diagnosis

As part of evaluating serum samples as a potential alternative matrix for rapid FAdV diagnosis, viral loads obtained from serum were compared with those measured in target tissues and environmental samples, including gizzard, liver, and cloacal swabs (CS or EC). This comparison aimed to assess the degree of concordance between serum viral loads and those obtained from organs across the four experimental groups.

#### 3.6.1 Overall correlation profiles

**Figure 9:**
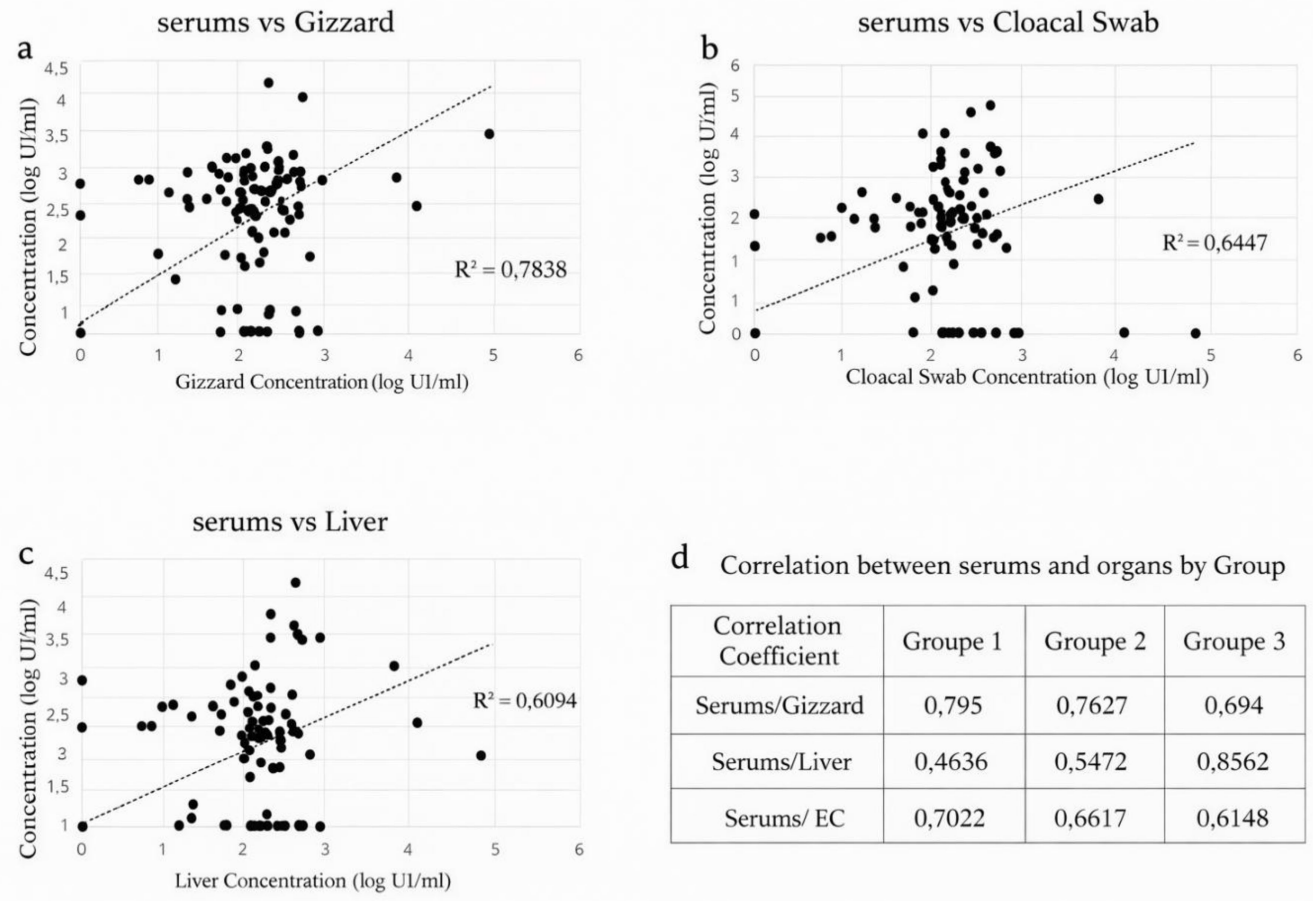
Correlation between serum viral loads and those measured in gizzard **(a)**, cloacal swabs **(b)**, and liver **(c)**. Panel **(d)** shows group-specific correlation coefficients between serum viral loads and other sample types.

Overall, the highest correlation was observed between serum and gizzard viral loads, with a mean correlation coefficient of R^2^ = 0.7838 across all groups (Figure 9a). In contrast, the correlation between serum and liver viral loads was lower (R^2^ = 0.6094) (Figure 9c), while an intermediate correlation was observed between serum and cloacal swabs (R^2^ = 0.6447) (Figure 9b). These results indicate a strong overall agreement between serum viral loads and those detected in major target tissues, particularly the gizzard.

##### 3.6.1.1 Group-specific correlation profiles

For Group 1, infected with FAdV serotype 1, the strongest correlation was observed between serum and gizzard viral loads (R^2^ = 0.795), followed by cloacal swabs (R^2^ = 0.7022). The weakest correlation was detected between serum and liver viral loads (R^2^ = 0.4636). Similarly, Group 2, also infected with serotype 1, showed the highest correlation between serum and gizzard (R^2^ = 0.7627), followed by cloacal swabs (R^2^ = 0.6617), and then liver (R^2^ = 0.5472). In contrast, Group 3, infected with FAdV serotype 8a, displayed a markedly different pattern. The highest correlation was observed between serum and liver viral loads (R^2^ = 0.8562), representing the strongest correlation among all comparisons performed. This was followed by gizzard (R^2^ = 0.694) and cloacal swabs (R^2^ = 0.6148) (Figure 9d).

Taken together, these results demonstrate that serum viral loads are strongly correlated with viral loads in target tissues, with correlation patterns varying according to the infecting FAdV serotype and its tissue tropism. For Groups 1 and 2, infected with serotype 1, serum viral loads were more closely associated with gizzard viral loads, whereas for Group 3, infected with the hepatotropic serotype 8a, serum viral loads were most strongly correlated with liver viral loads.

##### 3.6.1.2 Overall distribution of viral loads by sample type

**Figure 10:**
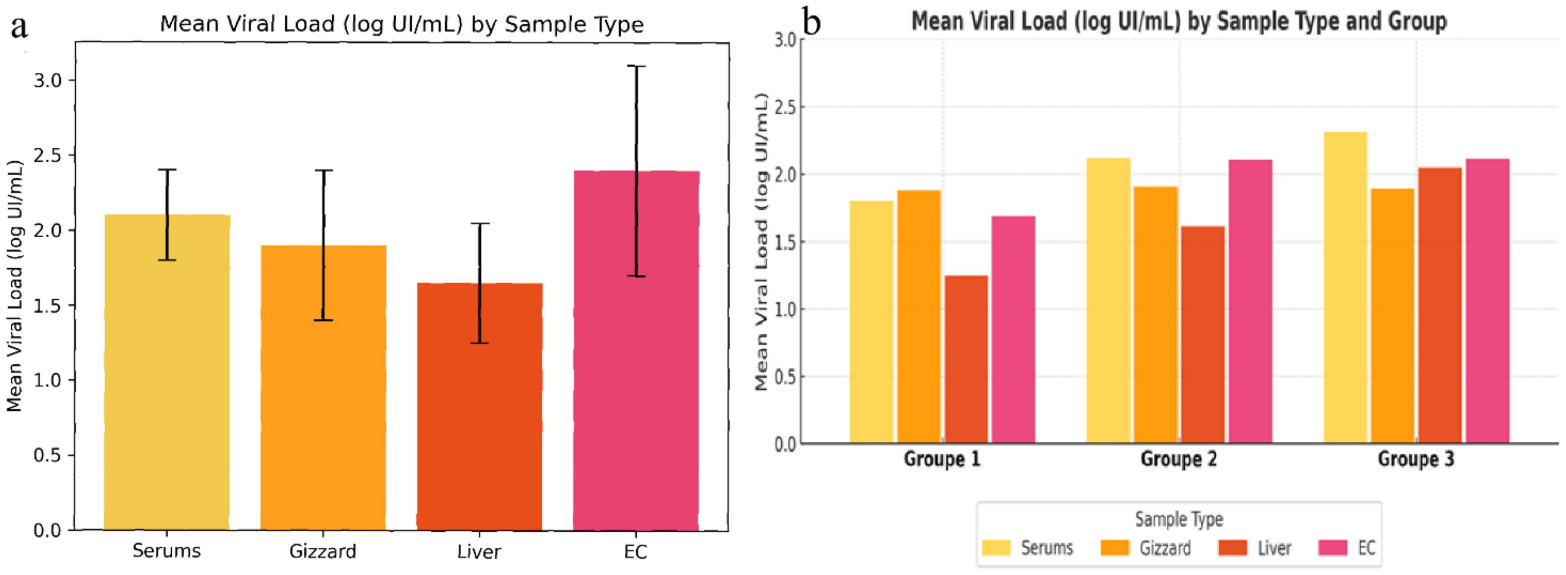
Mean viral load (log IU/mL) according to sample type and experimental group. **(a)** Overall mean viral loads measured in serum, gizzard, liver, and cloacal swab (EC) samples, pooled across all groups and sampling times. **(b)** Group-specific mean viral loads for each sample type in Groups 1, 2, and 3, illustrating differences in viral load distribution among sample matrices within each experimental group.

The overall distribution of viral loads by sample type is presented in Figure 10a. This analysis summarizes the meaning viral loads (log IU/mL) obtained from serum, gizzard, liver, and cloacal swab samples by pooling data from all experimental groups over the 30-day trial period. Among the different sample types, cloacal swabs exhibited the highest mean viral load, followed by serum, gizzard, and liver, indicating differences in viral distribution depending on the sampling matrix.

##### 3.6.1.3 Group-specific distribution of viral loads by sample type

The group-specific distribution of viral loads is illustrated in Figure 10b, providing a detailed comparison of viral load patterns within Groups 1, 2, and 3 over the 30-day experimental period. For each group, the reported values represent cumulative viral loads obtained for each sample type collected throughout the experiment. In Group 1, comparable viral loads were observed in serum and gizzard samples, whereas lower levels were detected in liver samples, with intermediate values in cloacal swabs, consistent with the non-hepatotropic nature of serotype 1. In contrast, Group 2 showed higher viral loads across all sample types compared to Group 1. This overall increase in viral loads in older birds suggests an age-related effect on viral replication dynamics, with 35-day-old chickens supporting higher levels of viral replication than younger birds.

Group 3, infected with FAdV serotype 8a, exhibited the highest viral loads overall and a distinct distribution pattern. In this group, serum samples showed the highest viral loads, slightly exceeding those detected in liver samples, followed by cloacal swabs and gizzard samples. The elevated liver viral loads confirm the hepatotropic nature of FAdV-8a, At the same time, the substantial viral loads detected in gizzard samples indicate a broader tissue distribution, consistent with the dual tropism of FAdV-8a.

##### 3.6.1.4 Overall evolution of mean viral loads by sample type

**Figure 11:**
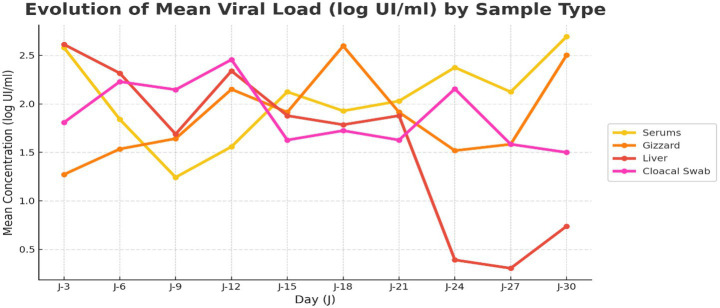
Temporal evolution of mean viral loads (log IU/mL) measured in serum, gizzard, liver, and cloacal swab samples over the 30 day experimental period. Viral loads were recorded every 3 days, illustrating time-dependent variations in viral detection across sample types.

Figure 11 illustrates the temporal evolution of mean viral loads (log IU/mL) measured in serum, gizzard, liver, and cloacal swab samples over the 30-day experimental period. Viral loads were monitored at regular time points (every 3 days), allowing comparison of viral dynamics among the different sample types during the assay.

Overall, serum viral loads showed relatively consistent detection over time, with a decrease between days 3 and 9, followed by a progressive increase from day 9 to day 30, reaching the highest viral load compared to the other sample types at the end of the experimental period. Gizzard samples exhibited pronounced variability, starting with the lowest viral load at day 3 and increasing over time, with a marked peak on day 18 and noticeable fluctuations at days 18 and 24, before reaching higher levels at day 30 after serum. Liver viral loads were initially higher but declined substantially after day 21, reaching the lowest levels toward the end of the experiment. In contrast, cloacal swab samples displayed moderate fluctuations over time, with a progressive decrease in viral load from day 12 until the end of the experimental period. These temporal trends indicate that viral load kinetics differ according to sample type, with serum and gizzard showing increasing detection at later time points, while liver and cloacal swab samples exhibit a progressive decline.

#### 3.6.2 Group-specific temporal evolution of mean viral loads by sample type

**Figure 12:**
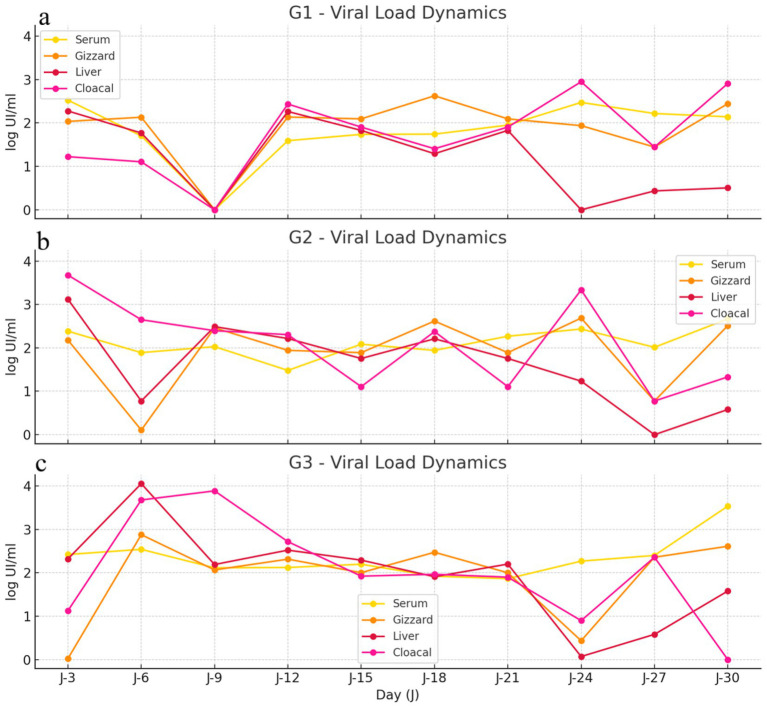
Group-specific viral load dynamics (log IU/mL) measured in serum, gizzard, liver, and cloacal samples over the 30 day experimental period. Panels **(a–c)** correspond to Groups 1, 2, and 3, respectively. Viral loads were monitored every 3 days, illustrating temporal variations in viral distribution across sample types within each experimental group.

In Group 1, viral loads were detectable in all sample types throughout the experimental period. All samples showed an initial decrease in viral load up to day 9, followed by distinct temporal patterns depending on the sample type. Serum viral loads exhibited moderate fluctuations and then progressively stabilized at mid and late time points. Gizzard viral loads followed a similar trend, remaining detectable throughout the assay with moderate variability. Liver viral loads were consistently lower than those observed in other sample types and showed a marked decrease after day 21, reaching minimal levels toward the end of the experiment. In contrast, cloacal samples displayed intermittent peaks, particularly at mid and late stages of the experiment, reaching the highest viral loads at the end of the experimental period. The viral load profile is consistent with a serotype 1associated non-hepatotropic pattern, with lower liver involvement and sustained detection in serum, gizzard, and cloacal samples over time (Figure 12a).

In Group 2, viral loads were detectable in all sample types throughout the experimental period. An initial decrease in viral load was observed up to day 6 in most sample types, followed by pronounced temporal fluctuations over the remainder of the assay. Serum viral loads showed moderate variability with a progressive increase at later time points. Gizzard samples exhibited marked fluctuations, including a sharp decrease at early time points followed by recovery at mid and late stages. Liver viral loads decreased progressively after mid-experiment, reaching very low levels toward the end of the experimental period. Cloacal samples displayed variable viral loads with distinct peaks at early and mid time points, followed by reduced levels at later stages. The higher variability across sample types suggests serotype 1 driven replication and shedding with dynamic kinetics, while liver viral loads remain comparatively low and tend to decrease toward later stages (Figure 12b).

In Group 3, infected with FAdV-8a, viral load dynamics reflected the dual tissue tropism of this serotype. High viral loads were observed in the liver at early point, indicating primary hepatic replication. These liver viral loads progressively declined toward the later stages of infection. In contrast, gizzard viral loads showed a gradual increase over time, consistent with the gastrointestinal involvement of FAdV-8a. Cloacal swab samples followed a pattern like that of the liver, with higher viral loads early in the experiment and a subsequent decrease. Notably, serum viral loads increased at later time points, particularly after day 27. This inverse relationship between declining liver viral loads and rising serum viral loads suggests a redistribution of viral particles from hepatic tissue into the bloodstream, accompanied by increased systemic dissemination and shedding, as reflected in cloacal swab samples. Together, these findings support a model in which FAdV-8a initially replicates in visceral organs, particularly the liver, followed by a late-phase systemic spread toward serum and excretory routes (Figure 12c).

## 4 Discussion

A universal real-time PCR assay targeting a conserved region of the FAdV penton gene was successfully developed and analytically and clinically validated. The selection of the penton gene as a molecular target was guided by whole-genome alignment of twelve reference FAdV serotypes, which identified a highly conserved region suitable for universal detection while preserving specificity across genetically diverse strains.

The assay demonstrated excellent analytical performance, as reflected by strong linearity over a wide dynamic range (10^2^–10^6^ copies/μL), a high amplification efficiency (97%), and a robust coefficient of determination (R^2^ = 0.9551). These parameters indicate reliable quantification across clinically relevant viral loads and are comparable tothe one developed by (23, 24), or exceed, those reported for previously described universal FAdV assays targeting the 52 K or fiber genes. The incorporation of degenerate bases in the forward primer design further ensured broad serotype coverage, effectively accounting for inter-serotype sequence variability without compromising assay efficiency. Specificity testing confirmed exclusive amplification of FAdV DNA, with no cross-reactivity observed against other major avian viral pathogens, including avian influenza virus, Newcastle disease virus, infectious bronchitis virus, and infectious laryngotracheitis virus. This high specificity is essential for routine diagnostic use, particularly under field conditions where co-circulation and co-infections with multiple avian viruses are common. Assay specificity was further supported by HRM analysis, in which five serial dilutions tested in duplicate from the same isolate consistently produced a single, well-defined melting peak, confirming the absence of non-specific amplification (21, 26).

Clinical validation was performed using 56 field samples collected from suspected cases of inclusion body hepatitis and adenoviral gizzard erosion. The assay successfully detected all FAdV-positive samples, including FAdV-8b and FAdV-11 from IBH cases and FAdV-8a and FAdV-1 from AGE cases. These results highlight the assay’s suitability for routine diagnosis as well as its potential utility in epidemiological surveillance and outbreak investigations. When compared with the reference universal real-time PCR/52 K assay described by Günes et al. (23), the penton-based assay demonstrated a strong quantitative correlation (R^2^ = 0.9762), as confirmed by linear regression analysis.

The clinical performance of the newly developed penton-based real-time PCR assay was further evaluated using a large panel of 320 experimental samples collected from chickens experimentally infected with FAdV-1 and FAdV-8a. These samples represented four distinct biological matrices, including serum, cloacal swabs, liver, and gizzard, allowing a comprehensive assessment of assay performance under conditions reflecting routine diagnostic practice and diverse tissue origins.

All samples were tested in parallel using both the reference universal real-time PCR/52 K assay and the newly developed penton-based assay, and viral loads obtained with the two methods were compared to assess quantitative concordance. Regression analysis revealed a strong positive correlation between the two assays across all sample types and serotypes, with a coefficient of determination of R^2^ = 0.8736, demonstrating that the penton-based assay reliably reproduces the quantitative behavior of the reference method regardless of matrix origin. In addition to regression analysis, comparison of mean viral loads across the 320 samples showed that the reference 52 K assay yielded slightly higher average concentrations than the penton-based assay. The limited dispersion observed between the assays further confirms the reproducibility and stability of viral load quantification achieved with the penton-based assay.

Together, these findings confirm the robust analytical and clinical performance of the penton-based real-time PCR assay, supported by its good linearity, high sensitivity and specificity, and strong quantitative concordance with the reference 52 K assay. The assay accurately detected all 56 FAdV-positive field isolates, representing four circulating serotypes and four sample matrices.

Secondly, the central objective of this study was to assess the suitability of serum as an alternative matrix for rapid FAdV diagnosis. To address this objective, chickens experimentally infected with FAdV-1 or FAdV-8a were monitored over a 30-day, and viral loads measured in serum were systematically compared with those obtained from gizzard, liver, and cloacal swab samples. This experimental design allowed the evaluation of serum performance across different tissue tropisms, infection dynamics, and host-related factors.

Overall correlation analyses revealed that serum viral loads were strongly associated with those measured in target tissues and cloacal swabs, with the highest correlation observed between serum and gizzard (R^2^ = 0.7838), followed by cloacal swabs (R^2^ = 0.6447) and liver (R^2^ = 0.6094). These findings indicate that serum viral loads reliably reflect systemic viral burden as well as tissue-level infection, particularly in organs involved in viral replication or persistence.

When analyzed by experimental groups, distinct correlation profiles emerged that closely matched the known biological behavior of the infecting serotypes. In Groups 1 and 2, both infected with FAdV serotype 1, serum viral loads showed the strongest correlation with gizzard samples, followed by cloacal swabs, while correlations with liver were consistently lower. This pattern is consistent with the non-hepatotropic nature of serotype 1 and its established association with adenoviral gizzard erosion only. In contrast, Group 3, infected with FAdV serotype 8a, exhibited a markedly different correlation profile. The strongest correlation was observed between serum and liver viral loads (R^2^ = 0.8562). These results demonstrate that correlation patterns are strongly influenced by the infecting serotype. The strong serum-liver and serum-gizzard correlations highlight the ability of serum viral loads to accurately reflect viral replication in both hepatic and gizzard tissues, depending on serotype-specific tissue tropism.

Analysis of mean viral loads across sample types provided additional insights into viral distribution. When data from all groups were pooled, cloacal swabs exhibited the highest mean viral loads, followed by serum, gizzard, and liver. This finding highlights the importance of cloacal shedding in FAdV transmission, while also demonstrating that serum consistently captures a substantial proportion of the overall viral burden. At the group level, Groups 1 and 2 (serotype 1) showed higher viral loads in serum and gizzard than in liver. Notably, Group 2, consisting of 35-day-old chickens, exhibited more pronounced viral loads than Group 1, suggesting that older birds may support more active viral replication at the gizzard level. In contrast, Group 3 (serotype 8a) showed elevated viral loads in both serum and liver, supporting the dual tropism of FAdV-8a for hepatic and gizzard tissues.

Group-specific temporal analyses further emphasized the combined influence of serotype and host age on viral kinetics. In Groups 1 and 2 (serotype 1), viral loads in serum, gizzard, and cloacal swabs remained detectable throughout the experiment, while liver viral loads remained low and progressively declined, consistent with a non-hepatotropic infection profile. Differences observed between Groups 1 and 2, despite infection with the same serotype, likely reflect age-related factors, as Group 1 consisted of 10-day-old chickens whereas Group 2 included 35-day-old birds, with potential differences in immune maturity and viral replication dynamics. In Group 3, infected with FAdV serotype 8a, viral load kinetics revealed a distinct temporal pattern consistent with the dual tropism of this serotype. High viral loads were observed in the liver at early time points, indicating primary hepatic replication, followed by a progressive decline toward the later stages of infection. In parallel, viral loads in gizzard samples increased over time, highlighting active gastrointestinal involvement. Serum viral loads, initially low, increased markedly during the late phase of the experiment, particularly from day 27 onward, accompanied by similar trends in cloacal swabs. This redistribution of viral loads suggests a late-phase dissemination of viruses from visceral tissues, particularly the liver, into the bloodstream and excretory routes. Together, these dynamics reflect the hepatotropic nature of FAdV-8a combined with increased gastrointestinal involvement, supporting its well-recognized dual tropism.

Taking together, these results demonstrate that serum is a valuable and reliable alternative sample for FAdV molecular diagnosis. Serum viral loads showed strong, serotype-dependent correlations with tissue viral loads and captured key temporal changes in viral dissemination during infection. Importantly, the interpretation of serum-based results should consider both the infecting serotype and host-related factors, such as age, which significantly influence viral distribution and infection kinetics. In contrast to tissue-based samples, which require multiple processing steps and are subject to variability related to tissue integrity and heterogeneous viral distribution, serum provides a simplified and standardized matrix for molecular testing. Serum-based analysis relies on a straightforward workflow involving blood collection and centrifugation, allowing the use of a defined input volume for DNA extraction. This standardization facilitates reproducible viral load quantification across samples and experimental groups and reduces technical variability. Together with the strong clinical correlations observed in this study, these practical advantages support the use of serum for routine molecular diagnosis, large-scale surveillance, and longitudinal monitoring of FAdV infections.

## Data Availability

The raw data supporting the conclusions of this article will be made available by the authors, without undue reservation.
